# Medical Cannabis and the Hallmarks of Cancer: A Critical Narrative Review

**DOI:** 10.3390/ijms27177549

**Published:** 2026-08-23

**Authors:** Diana Russo, Rute Fernandes, Valéria Tavares, Ana Agrelo, Rui Medeiros

**Affiliations:** 1 Portuguese Oncology Institute of Porto (IPO Porto), 4200-072 Porto, Portugal; 2 Molecular Oncology and Viral Pathology Group, IPO Porto Research Centre (CI-IPOP), Department of Pathology and Laboratory Medicine, RISE-Associate Laboratory (Health Research Network), Porto Comprehensive Cancer Centre Raquel Seruca (Porto.CCC), Portuguese Oncology Institute of Porto (IPO Porto), 4200-072 Porto, Portugal; 3 School of Medicine and Biomedical Sciences (EMCB), Fernando Pessoa University, 4420-096 Gondomar, Portugal; 4 Research Department, Portuguese League Against Cancer (NRNorte), 4200-172 Porto, Portugal; 5 Faculty of Health Sciences, Fernando Pessoa University, 4200-150 Porto, Portugal; 6 Instituto de Ciências Biomédicas Abel Salazar (ICBAS), University of Porto, 4050-313 Porto, Portugal; 7 European Cancer Organisation (ECO), 1040 Brussels, Belgium

**Keywords:** cannabinoids, cannabidiol, tetrahydrocannabinol, signal transduction, tumour microenvironment, drug therapy, combination

## Abstract

Cancer remains a highly complex and heterogeneous disease, causing major morbidity and mortality worldwide despite advances in diagnosis and treatment. The hallmarks of cancer enlighten the biological mechanisms supporting tumourigenesis and malignant evolution, while helping to identify potential therapeutic targets. Medical cannabis has mostly been used in oncology for supportive care, but increasing preclinical evidence suggests interference with cancer-related signalling pathways. This narrative review summarizes the current evidence on cannabinoids, in particular the phytocannabinoids cannabidiol (CBD) and Δ9-tetrahydrocannabinol (THC), framing their prospective anticancer effects in the hallmarks of cancer. Preclinical studies imply that CBD exerts antiproliferative effects by modulating oncogenic signalling pathways, including EGFR, PI3K/AKT, RAS/RAF/ERK, mTOR and Wnt/β-catenin, while also influencing tumour suppressor pathways involving p53, p21 and p27, causing cell cycle arrest. CBD has additionally been shown to promote programmed cell death via mitochondrial dysfunction and autophagy, altering cancer metabolism as well. Furthermore, CBD has shown anti-invasive and antiangiogenic properties and also appears to modulate immune responses and interactions with the tumour microenvironment, including emerging links with the microbiome. Overall, cannabinoids exhibit biologically plausible antitumour activity across multiple cancer hallmarks and may present promising candidates for combination therapeutic strategies. Nonetheless, the current evidence remains predominantly preclinical, and robust translational studies and clinical trials are needed to clarify their pharmacokinetic and pharmacodynamic profiles, determine their clinical efficacy and safety while assessing their potential integration into multimodal cancer treatment.

## 1. Introduction

Cancer is a leading cause of death worldwide, accounting for 20 million new cases annually worldwide and 10 million deaths with a devastating global burden [1]. Despite screening, diagnosis and systemic treatment advances, cancer remains characterized by remarkable biological complexity, therapeutic resistance and disease progression, posing major challenges in improving long-term patient outcomes.

The latest *Hallmarks of Cancer* provide a comprehensive conceptual framework for understanding the biological processes sustaining tumour initiation, progression and therapeutic resistance. These hallmarks include sustained proliferative signalling, resistance to growth suppression and cell death, replicative immortality, angiogenesis, invasion and metastasis, metabolic reprogramming, immune evasion and unlocking phenotypic plasticity. More recently, enabling characteristics and emerging dimensions of cancer biology, such as genomic instability/chromosomal instability, epigenetic reprogramming, tumour-promoting inflammation, polymorphic microbiomes and innervation have also been recognised as relevant dimensions of cancer biology [2].

Despite being commonly used for recreational purposes (mainly due to the psychoactive effects of Δ9-tetrahydrocannabinol), *Cannabis sativa* has been used medicinally for centuries, with documented therapeutic use dating back to at least 500BCE for pain management, nausea and glaucoma. The plant contains numerous natural compounds, including phytocannabinoids, terpenes and flavonoids, of which cannabidiol (CBD) and Δ9-tetrahydrocannabinol (THC) are the most comprehensively studied chemical components [3,4]. CBD has anti-inflammatory, antiemetic and anti-tumour effects, among others, while THC is the chief psychoactive component of *Cannabis sativa*, is recognised for its analgesic and antiemetic effects and has potential antitumour activity in experimental models [5].

Although structurally related, CBD and THC have distinct pharmacological profiles, with THC being a partial agonist of both cannabinoid receptor 1 (CB1R) and cannabinoid receptor 2 (CB2R) and its effects being strongly influenced by receptor expression and signalling efficiency in each tissue or tumour type, whereas CBD has low affinity for both receptors and can act as a negative modulator of CB1R [6]. Accordingly, THC-mediated anticancer effects are more frequently associated with CB1R/CB2R-dependent modulation of Mitogen-activated protein kinase (MAPK)/Extracellular signal-regulated kinase (ERK) and Phosphoinositide 3-kinase (PI3K)/AKT signalling, cell-cycle arrest, endoplasmic reticulum stress and autophagy, whereas CBD exhibits a broader multi-target profile involving Transient receptor potential vanilloid (TRPV) channels, G protein-coupled receptor 55 (GPR55) and peroxisome proliferator-activated receptor gamma (PPARγ), together with oxidative stress, mitochondrial dysfunction, apoptosis and inhibition of proliferative signalling pathways [5,7,8,9,10].

On the other hand, the endogenous cannabinoid system (ECS) is a widely distributed lipid-signalling network composed primarily of the cannabinoid receptors CB1R and CB2R, the endogenous ligands anandamide (AEA) and 2-arachidonoylglycerol (2-AG) as well as the enzymes responsible for their synthesis and breakdown [11]. CB1R is highly expressed in the central nervous system but is also found in several peripheral tissues, whereas CB2R is particularly abundant in immune and haematopoietic cells, albeit both receptors exhibit broader and context-dependent tissue distributions. The ECS acts as a homeostatic regulatory system that is recruited following physiological stress or tissue injury and contributes to the restoration of neural, endocrine, metabolic and immune balance. Several processes regulated by the ECS—including cellular metabolism, inflammatory signalling, immune-cell activity and responses to tissue injury—are also implicated in tumour development and progression, providing a biological rationale for investigating cannabinoid signalling in cancer [11,12].

In the oncology setting, cannabinoids are currently established as supportive care agents for the control of chemotherapy-induced nausea and vomiting, cancer-related pain and other treatment-associated symptoms. However, growing preclinical evidence suggests that cannabinoids may also influence cancer biology, by modulating multiple cellular and molecular processes involved in tumour progression. Preclinical studies suggest that CBD and other cannabinoids can modulate cell proliferation, induce cell cycle arrest, apoptosis and autophagy, inhibit tumour cell migration, invasion and angiogenesis, modulate immune and inflammatory responses, and enhance the activity of conventional anticancer therapies in selected experimental models [13,14]. These antitumour capacities have increasingly been gaining attention and have been demonstrated across a range of malignancies, including colorectal, lung, breast, ovarian and prostate cancer [13,14,15]. Although these findings support a biologically plausible role for cannabinoids as potential adjuncts in cancer therapy, the available evidence remains mostly preclinical and heterogeneous, requiring further clinical validation. Therefore, this narrative review aims to summarise the current evidence exclusively regarding the effects of cannabinoids on cancer biology and to contextualise these findings within the updated *Hallmarks of Cancer* framework, highlighting the molecular mechanisms through which these compounds may influence tumour progression and response to treatment (Table 1).

## 2. Replicative Immortality

Replicative immortality enables cancer cells to overcome the intrinsic limits of cell division, mostly by bypassing telomere shortening, replicative senescence and crisis. This is primarily achieved through telomerase reactivation or alternative lengthening of telomeres, allowing unlimited proliferative capacity [2]. Emerging evidence suggests that *Cannabis sativa* may interfere with several of these processes by modulating TP53 signalling, cell cycle regulation and the expression of genes involved in telomere maintenance [2,5].

Experimental evidence has shown the effects of CBD on breast cancer models, including MCF-7 cells [16,17]. These effects appear to be mediated through the cannabinoid receptor 1 (CB1R) and 2 (CB2R), as well as non-cannabinoid targets such as transient receptor potential vanilloid 1 (TRPV1), GPR55 and PPARγ [16]. These studies have shown a significant increase in the number of apoptotic cells in breast cancer cells treated with CBD, supporting its promotion of early and late-stage apoptosis [16,17]. Besides, to determine the selectivity of CBD toward cancer cells, the same concentrations were applied to CCD-1072Sk normal fibroblast cells, showing a markedly higher sensitivity of cancer cells to CBD treatment [16]. These findings suggest that CBD may affect not only cell survival, but also mechanisms of uncontrolled proliferation. Mechanistically, CBD increases TP53 protein expression and promotes cell-cycle arrest, with accumulation of cells in the G0/G1 and S phases, depending on the experimental model. In addition, CBD downregulates several genes involved in telomere structure and maintenance, including *TNKS2*, *PINX1*, *TINF2*, *TERF1*, *TEP1*, *TERF2IP* and *TNKS* [5,16]. As these proteins are essential for telomere integrity and telomerase regulation, their reduced expression may impair telomere maintenance, promote telomere shortening and ultimately restrict the replicative potential of tumour cells [16,17]. In MCF7 cells, CBD-mediated apoptosis and cytotoxicity have also been associated with decreased mitochondrial energy production, suggesting that metabolic disruption may further contribute to the loss of proliferative capacity [17].

Beyond its effects on telomere biology, CBD has been shown to inhibit the invasive and metastatic nature of triple-negative breast cancer while suppressing the activation of the epidermal growth factor(EGF)/epidermal growth factor receptor(EGFR) pathway. Compared to daily administration, however, CBD administered three times a week boosted the longevity of mice and reduced the number of metastases [17]. In cell line studies, CBD synergistically enhanced the antiproliferative activity of chemotherapeutic drugs by promoting apoptosis [17]. This compound inhibited forkhead box protein M1 (FOXM1), a cell cycle regulatory gene closely associated with neoplasm formation, while also facilitating growth differentiation factor 15(GDF15), contributing to tissue differentiation and inhibition of abnormal proliferation. These phenomena could be seen in glioblastoma (GBM), salivary duct, breast and prostate cancer [5].

Although the available evidence remains almost exclusively preclinical, current findings suggest that CBD may limit replicative immortality through coordinated effects on TP53 signalling, cell-cycle regulation, telomere maintenance and key drivers of uncontrolled proliferation [5,16,17]. Further mechanistic studies and clinical investigations are required to determine whether these findings translate into meaningful therapeutic benefit in patients.

## 3. Deregulation of Cellular Metabolism

Cancer cells can adjust their energy pathways to sustain continuous growth, proliferation and survival, by using oxidative phosphorylation and aerobic glycolysis, while depending on alternative fuels [2].The use of Phyto-cannabinoids is associated with a reduction of adenosine triphosphate (ATP) concentration in the cell, which is particularly relevant to the activation of the AMP-activated protein kinase (AMPK), a sensor of cellular energetic stress that promotes cellular adaptation to energetic stress and serves as an upstream regulator of autophagy (inhibiting mTOR) [18,19]. Therefore, *Cannabis sativa* may influence cancer metabolism by forcing tumour cells into an energetic stress state, ultimately affecting survival.

CBD and THC modulate multiple intracellular signalling pathways that regulate tumour cell metabolism, proliferation and survival. In GBM, CBD increases the phosphorylation of c-Jun N-terminal kinase (JNK1/2-P) and p38 mitogen-activated protein kinase (p38 MAPK) activities, while suppressing the nuclear factor kappa B (NF-κB) signalling, thereby promoting apoptosis and reducing cell viability [19]. The combined treatment of temozolomide and THC results in a higher activation of autophagy, leading to a markedly more effective tumour growth inhibition and antitumour effect [19]. Furthermore, CBD and THC combination lowered the levels of specific subunits of the respiratory chain complexes I and IV, impairing mitochondrial respiration in human GBM cells and leading to a diminished oxidative phosphorylation and ATP production [5]. In prostate cancer, CBD restricts the release of extracellular vesicles from cancer cells, disrupting mitochondrial respiration and energy production [20].

CBD also disrupts intracellular calcium homeostasis and endoplasmic reticulum function by activating transient receptor potential vanilloid 4 (TRPV4). This causes stress of the endoplasmic reticulum and affects the influx of calcium, while also altering the mitochondrial electron transport chain and respiration, thus inhibiting cellular metabolism and resulting in an anti-tumour effect [19]. Additionally CBD also acts on the transient receptor potential vanilloid 2 (TRPV2) signalling pathway, resulting in the activation of autophagy genes activation and increased expression of autophagy markers such as LC3-II and Beclin-1/class III phosphatidylinositol 3-kinase (PI3K-III) complexes [7]. Besides, in a study in patients with GBM those effects were not seen in normal human astrocytes, showcasing a potential selective effect of CBD on cancer cells [21]. Together, these mechanisms suggest that CBD may connect metabolic stress, calcium signalling, endoplasmic reticulum dysfunction and autophagy activation to promote cancer cell death. Furthermore, CBD regulates transmembrane signalling sensors of the endoplasmic reticulum (ER) surface, such as PERK and IRE1 kinases, BIP protein and activating transcription factor (ATF). Through mediation of the sensors in the ER, CBD enhances the ASK1(apoptosis signal-regulated kinase 1)/JNK axis while upregulating CCAAT/enhancer-binding protein homologous protein (CHOP) expression in the nucleus, leading to apoptosis. CBD-triggered ROS and ER stress were found to result in NOXA proapoptotic protein activation in colorectal cancer and ER stress can also result in mitochondrial malfunction. CBD treatment disrupts redox homeostasis while at the same time promoting ROS and ER stress, therefore speeding the apoptotic process [5]. This reinforces that CBD may impair tumour cell metabolism not only by targeting ATP production and mitochondrial respiration, but also by inducing redox imbalance and ER stress.

THC also promotes tumour cell death by targeting signalling pathways involved in cell survival. It suppresses the Rat sarcoma(RAS)-MAPK and PI3K-Protein kinase B (AKT) survival signalling pathways, leading to activation of the pro-apoptotic protein BAD, increased ROS production and caspase activation, culminating in apoptosis [12]. CB1R and CB2R are G protein-coupled receptors that inhibit adenylate cyclase following activation, reducing the formation of cyclic adenosine monophosphate, influencing the RAS/Rapidly accelerated fibrosarcoma(RAF)/Mitogen-activated protein kinase/extracellular(MEK)/ERK signalling cascade, a central regulator of cell proliferation, survival, differentiation and migration [22]. Through these effects, cannabinoids may indirectly impair the metabolic phenotype that supports tumour growth.

In pancreatic cancer cells, cannabinoids induce apoptosis through inhibition of pancreatic beta cell insulin receptor signalling, which influences the CB1R through the MAPK and ERK pathways [23]. Furthermore, CBD induces elevated BAX (from BCL-2 family) in a dose-dependent manner, while potentially promoting BID-t-BID conversion, disrupting the outer mitochondrial membrane [5].

## 4. Evasion of Immune Destruction

Cancer cells can escape immune system recognition and elimination, through suppression of adaptive and innate immune responses, namely via T cell and NK cell dysfunction, thus contributing to an immunosuppressive microenvironment [2]. In this setting, cannabinoids have shown important immunomodulatory effects that may either suppress or enhance immune-mediated tumour control, depending on the circumstance. CB2R, a target of CBD, is widely distributed throughout the immune system, being a potential regulator of the immune system [24]. Thus, modulation of CB2R may influence immune cell activation, cytokine production and the balance between immune suppression and activation. Some emerging evidence suggests cannabinoids can disrupt the functionality of tumour-targeting T cells while engaging with the CB2R, and caution is needed when using cannabinoids alongside immunotherapy [25].

Some emerging evidence suggests cannabinoids can disrupt the functionality of tumour-targeting T cells while engaging with CB2R, and caution is needed when using cannabinoids alongside immunotherapy. However, clinical findings remain inconsistent with some observational studies reporting lower response rates or poorer outcomes among cannabis users receiving ICIs [26,27], whereas other clinical cohorts and pooled trial data found no significant detrimental effect [28,29].

Myeloid-derived suppressor cells (MDSCs) are T cells suppressors and were shown to be triggered by CBD, resulting in decreased levels of pro-inflammatory factors such as IL-17 and IFN-γ, with an increase in IL-10 levels [30,31]. CBD also inhibits gasdermin-mediated pyroptosis in the gastrointestinal (GI) trac t [31]. These findings support a possibly immunosuppressive effect of CBD through inhibition of inflammatory cytokines and promotion of regulatory immune mechanisms.

THC induces apoptosis in dendritic cells, inhibiting immune responses, whilst CBD promotes a non-immunogenic phenotype, inducing TNF-α, IL-10 and IL-6 secretion and reducing T cell activation [32,33]. Furthermore, CBD-enhanced apoptosis has also been validated in T lymphocytes through oxidative stress and caspase release from the mitochondria [13]. CBD has shown to markedly reduce TNF-α, IL-10 and IL-6, suppressing the activity of RAW 264.7 monocytes and peritoneal macrophages [31]. On the other hand, CBD may induce a tolerogenic effect while sequestering T cells and diminishing IFN-γ secretion by obstructing transcription protein-1 (AP-1) activation and targeting the IFN-γ receptor directly, decreasing its expression [31,34].

Altogether, these mechanisms suggest that cannabinoids may contribute to immune evasion by reducing antigen presentation, T cell activation, macrophage inflammatory activity and pro-inflammatory cytokine secretion. However, considering CBD’s suppression of immunostimulatory cells and cytokines was contradictory to its antitumour activities, additional experiments showed the complexity of CBD immunomodulation. CBD showed dose dependent influence on immune cells, decreasing B, T, T helper and T cytotoxic lymphocyte subsets at doses of 5 mg/kg/day, while increasing the counts and percentages of NK and NKT cells at a dose of 2.5mg/kg/day, implying a nonspecific immune response [35]. CB2R is strongly expressed in cytokine-induced killer cells (CIK) and CBD is protective in these cells in a dose-dependent manner with high concentrations reducing the CIK cells’ cytotoxic effect on multiple myeloma cells [36]. Moreover, CBD increased the cytotoxicity of CIK cells and reduced the activity of PANC-1 cells in pancreatic cancer in a significant way [37]. CBD enhanced the proliferation of T-Cells and decreased GBM-induced lymphopenia in a murine model, contributing to an immunostimulatory environment and diminished tumour growth [19]. Another study in GBM showed that CBD inhalation treatment also blocks 2,3-dioxygenase (IDO) therefore upregulating CD8+ T cells’ proportion and the expression of CD103, facilitating antigen presentation and tumour killing effects [38]. These results suggest that under different specific conditions, CBD counteracts tumour immune evasion while improving T cell proliferation, increasing cytotoxic immune subsets and enhancing antigen presentation.

In conclusion, CBD appears to exert context-dependent immunomodulatory effects, with its impact varying according to concentration, immune cell subtype and tumour context. Current evidence suggests that lower concentrations may be more favourable for promoting antitumour immune responses, whereas higher concentrations may exert predominantly immunosuppressive effects. Overall, cannabinoids demonstrate a complex and bidirectional influence on the immune system, indicating that their effects on immune evasion and tumour immune surveillance are highly dependent on the biological and experimental context.

## 5. Activation of Invasion and Metastasis

Tumour cells can invade neighboring tissues, enter blood and lymphatic vessels, moving to distant organs, adapting to new tissue microenvironments, forming the metastatic cascade. This process is responsible for much of cancer morbidity and mortality [2]. Cannabinoids, particularly CBD, have shown anti-invasive and anti-metastatic properties, modulating extracellular matrix degradation, epithelial-mesenchymal transition (EMT), cell adhesion and migration. The invasion cascade then culminates in metastases involving the dissemination of tumour cells to distant organs and adaptation to a foreign tissue microenvironment [5].

Cannabinoids seem disrupt proliferation on tumours by inhibiting matrix metalloproteinases (MMPs) whilst affecting metastasis through modulation of extracellular matrix degradation, resulting in decreased invasion [23]. This suggests cannabinoids may interfere with early steps of local invasion.

In breast cancer cell models, CBD has reduced the secretion of IL-1β, reversing EMT [39]. Consistently, when using CBD in tumour cells the level of AKT phosphorylation reduced, silencing the IL-1β/IL-1R/β-catenin pathway [5]. Since EMT and β-catenin signalling are important mechanisms for metastatic dissemination, this supports a potential anti-metastatic role of CBD in breast cancer.

In colorectal cancer cells, CBD has been demonstrated to increase E-cadherin and reduce N-cadherin expression, blocking the Wnt/β-catenin signalling pathway and inhibiting the formation of EMT, preventing cancer cell metastasis [40]. Besides, the CBD-mediated inhibition of the GPR55-PI axis impairs colon tumour cells-endothelial cells adhesion and the GPR55 silencing prevents cancer cell migration and invasion [8]. These findings suggest that CBD might reduce metastatic potential by reversing EMT as well as impairing tumour-endothelial adhesion, a key step for extravasation and metastasis.

In GBM cells, CBD has also reduced prohibitin (PHB) levels, a protein that positively regulates E-cadherin expression and relates to poor prognosis [41]. In a melanoma cell model, a study exhibited that the activation of CB2R on cerebral endothelial cells results in a reduced adhesion and transmigration of melanoma cells, making them a potential target for melanoma metastasis inhibition [42].

Furthermore, in a lung cancer cell model, CBD decreases the expression levels of inhibitor of DNA binding protein-1 (ID-1) which is amplified in highly aggressive cancer cells and is a major gene mediating breast cancer metastasis to the lung [43,44]. CBD also attenuates plasminogen activator inhibitor 1 (PAI-1) levels in lung cancer cells, accounting for the anti-invasive effect [45]. Besides, CBD also increases Intercellular adhesion molecule 1 (ICAM-1) expression and downregulates EGF through cannabinoid receptors and TRPV1, mediating an anti-invasive effect dependent on tissue inhibitor of metalloproteinase-1 (TIMP-1) and MMP [46,47]. These mechanisms reinforce CBD’s role in regulating invasion-associated pathways, including EGF signalling, ID-1 expression and balancing between MMPs and their inhibitors.

Studies have reported that deregulation of endocannabinoids during carcinogenesis results in increased cancer aggression [23]. Evidence suggests that cannabis may influence invasion and metastasis via multiple, complementary mechanisms, namely EMT inhibition, modulation of Wnt/β-catenin and IL-1β/AKT signalling, MMP-related extracellular matrix degradation and suppression of pro-metastatic mediators.

## 6. Phenotypic Plasticity

Cancer cells can both change their differentiation status and acquire new cellular phenotypes, enabling adaptation to diverse environments, metastasis, therapy resistance and ultimate survival. These changes can occur via intralineage plasticity or even cancer stem cells (CSC) [2]. *Cannabis sativa* can modulate CSCs and their signalling mechanisms, as well as differentiation pathways. These cells have a high self-renewal potential, possessing tumourigenic, drug-resistance and differentiating characteristics, closely related to tumour heterogeneity, recurrence and resistance to therapy [48]. CBD can regulate signalling pathways related to CSCs, as in triple-negative breast cancer (TNBC), affecting Janus kinase-signal transducer (JAK-STAT) and Wnt/β-catenin signalling pathways, as well as ER stress [49,50]. CBD also inhibits the Src/Von Hippel-Lindau tumour suppressor(VHL)/Hypoxia-inducible factor 1 alpha(HIF-1α) signalling contributing to suppressing breast cancer recurrence and metastasis [51]. Importantly, in the same study, CBD reduced proliferation across breast cancer cells at higher concentrations, while no comparable reduction was observed in non-tumorigenic MCF10A mammary epithelial cells, suggesting relative tumour selectivity in this model [51].These pathways are involved in stemness maintenance and tumour adaptation, making them relevant for the cancer cells’ metastatic potential.

In lung cancer, CBD inhalation reduces CD44, a cell adhesion molecule, highly expressed in CSCs and relates to progression and metastatic potential [52]. In GBM, CBD exerts selective cytotoxicity towards CSCs through GPR55 and TRPV1, with greater toxicity observed in CSCs than in differentiated cells [53]. This preferential targeting suggests that CBD may be particularly effective against the highly plastic tumour cell populations that drive disease progression and treatment resistance.

Beyond its direct cytotoxic effects, CBD also disrupts the interactions between CSCs and the tumour microenvironment (TME), thereby diminishing the tumour aggressiveness and enhancing the efficacy of conventional anticancer treatments [54]. By simultaneously targeting CSC survival and weakening the supportive signals provided by the TME, CVD may limit phenotypic plasticity and reduce tumours’ capacity to adapt to therapeutic pressure.

The activation of cannabinoid receptors alters the expression of regulatory genes related to stem cell proliferation and differentiation, contributing to the inhibition of tumourigenesis of CSCs [54]. GBM tumours contain a subpopulation of cells that can form tumour-neurospheres (a functional marker of stem-cell behaviour), and treatment of CSCs with sub-lethal concentrations of cannabis may inhibit neurosphere formation showing up to 60% [43,55].

The Wnt/β-catenin pathway relates intimately to drug resistance present in the majority of CSCs, such as ovarian cancer. T his pathway is reduced by CBD, which also reduces CD44 levels and the NFkB pathway, both relevant for CSC development [56,57]. CBD also upregulates Nrf2, which regulates the cell antioxidant system associated with neuroprotection, activated in CSCs, playing an important role in tumour initiation, metastasis and drug resistance [58]. CBD also promotes AML1a-dependent CSC differentiation, contributing to autophagy [57].

Collectively, the available evidence thus suggests that CBD could influence phenotypic plasticity by targeting CSC survival, reducing stemness markers (such as CD44), inhibiting pathways like Wnt/β-catenin and NFkB, and overall affecting tumour cell adaptation mechanisms.

## 7. Induction or Access to Vasculature

Cancer can to secure blood supply and maintain oxygen and nutrient delivery, therefore sustaining continuous tumour growth, mostly attained through angiogenesis. This process of forming new vessels is driven by hypoxia-induced proangiogenic factors like vascular endothelial growth factor A (VEGFA) and AGTP2, contributing to abnormal and often dysfunctional tumour vessels [2]. Tumours release angiogenic factors to promote the formation of blood vessels during progression, resulting in an imbalance of angiogenesis inducers and inhibitors [5].

Cannabinoids, mainly CBD, have shown great antiangiogenic potential, reducing endothelial cell migration and the expression of angiogenesis-related genes. CBD suppresses human umbilical vein endothelial cell (HUVEC) migration in lung cancer cells, through upregulation of the TIMP-1, preventing migration and angiogenesis, which was not seen in CBD-treated normal bronchial epithelial tissue [59]. TIMP-1 is also an endogenous suppressor of metalloproteinases, and inhibits MMP family members’ activity, suggesting that CBD may selectively reduce tumour-associated endothelial migration and extracellular matrix remodelling, without the same effect being observed in normal bronchial epithelial tissue [59].

Studies showed that CBD diminished various proangiogenic factors such as MMP2 and 9, IL-8, endothelin-1 (ET-1),CXC chemokine ligand 16 (CXCL16) and platelet-derived growth factor AA (PDGF-AA), altering the expression of such angiogenic proteins in HUVECs [59].

CBD reduces the expression of several angiogenesis-related genes including *ANGPT2*, *FLT1* and *KDR*. In MCF-7/Adr cells, CBD treatment decreases angiogenesis-related genes *FLT1* and *TEK* expression, while increasing apoptosis-related genes *APAF1*, *CASP9* and *BIRC3* expression [16]. Therefore, CBD may simultaneously suppress proangiogenic signalling and promote apoptosis-related pathways.

Whilst in murine gestational models THC decreases offspring blood vessel density and vascular endothelial growth factor (VEGF), in colorectal cell lines it appears to increase VEGF-A protein, while CBD has shown to decrease vascular structure, vessel size and VEGF protein expression in xenografts of human lung cancer (on 6.4 mg/day) [25]. Further reinforcing that cannabinoids may affect VEGF-dependent angiogenesis, one of the central mechanisms involved in tumour neovascularisation.

GPR55 is a transmembrane protein related to cannabinoids and influences endothelial cell proliferation by reducing nerve growth factor (NGF) levels in the TME, which might be a route through which cannabinoids modulate angiogenesis and vascular integrity [5]. Thus, the evidence suggests cannabinoids might interfere with both the induction and maintenance of tumour vasculature, by decreasing endothelial migration, VEGF expression, angiogenesis-related genes, MMP activity, as well as proangiogenic cytokines, while also potentially modulating vascular integrity through GPR55-related mechanisms.

## 8. Resistance to Programmed Cell Death

Cancer can escape mechanisms designed to eliminate damaged or abnormal cells, namely apoptosis, which is the main way for programmed cell death. Other mechanisms such as autophagy, necroptosis and even pyroptosis may also contribute to cancer cell elimination, but are all avoided by tumour cells thus resulting in their survival [2]. Cannabinoids, particularly CBD, have been shown to increase oxidative stress and disrupt mitochondrial function, therefore contributing to cancer cells’ apoptosis. CBD contributes to the upregulation of NADPH oxidases Nox4 and p22phox, leading to the death of cancer cells, by enhancing apoptotic effectors such as BAX, CASP3 and CASP9, disrupting mitochondrial function [31,60].

In hepatocellular carcinoma (HCC) cell lines, CBD induces mitochondrial-mediated apoptosis, reducing Bcl-2 and increasing BAX, CASP3,8 and 9 expression, as well as inducing G0/1 cell cycle arrest. Clinical observations showed that the high expression of CB1/2 receptors correlates with improved disease-free survival in HCC [61,62]. This way, CBD may promote apoptosis in HCC by modulating the anti-apoptotic and pro-apoptotic protein balance.

In endometrial cancer, CBD diminishes the migration and proliferation of Ishikawa cells by increasing ROS levels and activating the mitochondrial apoptotic pathway, resulting in cell death by activating autophagy [63]. This raises the question that CBD might induce overlapping programmed cell death mechanisms through the connection of oxidative stress, mitochondrial apoptosis and autophagy-related pathways.

Recent studies have shown that a high-THC cannabis strain had an important cytotoxic impact on human GBM cell lines, inducing ER-stress and apoptosis [55]. Overall, these findings support the cannabinoids’ role in promoting programmed cell death, mainly through apoptosis and endoplasmic reticulum stress, while also counteracting resistance to programmed cell death by increasing ROS production, activating mitochondrial apoptosis and modulating the Bcl-2/BAX balance.

## 9. Sustained Proliferative Signalling

Cancer cells can maintain a continuous activation of growth and cell cycle pathways, mostly through oncogenes such as *KRAS*, *PIK3CA*, *BRAF*, *EGFR*, *HER2*, *MYC* and *CCND1*, with altered gene expressions allowing tumour cells to bypass normal proliferative control and sustain continuous division and proliferation [2]. In this context, CBD has shown antiproliferative effects on several tumour models. Namely, it downregulates EGFR and inhibits pathways such as PI3K/AKT, RAS/rapidly accelerated fibrosarcoma (RAF)/ERK, and p53, associated with tumour proliferation and differentiation [5]. As EGFR, PI3K/AKT and RAS/RAF/ERK are central pathways involved in growth factor signalling and cell cycle progression, CBD-mediated inhibition of these mechanisms may contribute to reduced tumour proliferation.

The combination of CBD and THC effectively inhibit proliferation in paediatric brain cancer models [64]. In breast cancer cell lines, CBD demonstrated synergy with conventional therapies, with the combination of CBD and somatostatin significantly altering cell membrane receptor expression, reducing proliferation [17]. In TNBC, CBD downregulated mTOR and cyclin D1 signalling and enhanced PPARγ activity, repressing proliferation [9]. In ovarian cancer cells, CBD inhibits PI3K/AKT signalling, enhancing cisplatin sensitivity [56]. Furthermore, both CBD and THC showed preferential cytotoxicity toward A2780 and SKOV3 ovarian cancer cells compared with non-tumorigenic IOSE80 ovarian epithelial cells, with approximately four- to five-fold higher IC_50_ values in the non-malignant cells, with their combination enhancing cell-cycle arrest and apoptosis in the cancer cells [65]. Therefore, cannabinoids might exert direct antiproliferative effects while also increasing the activity of other anticancer treatments. At the same time, in gastric cancer models, CBD at 10μM induces apoptosis by triggering endoplasmic reticulum stress and at 20–40 μM/mL inhibits cell proliferation and the estabelishment of colonies while inducing G0-1 cell cycle arrest [3]. This reinforces the role of CBD in limiting proliferative expansion through both cell cycle arrest and activation of cell death mechanisms. Additionally, FOXM1 is suppressed by CBD and GDF15 is induced, thus inhibiting uncontrolled cell division [66].

Overall, CBD and other cannabinoids may interfere with sustained proliferative signalling by targeting several pathways while also promoting differentiation, treatment sensitivity and enhancing cell cycle progression.

## 10. Inactivation of Growth Suppressors

Cancer cells can inactivate growth suppressors responsible for normally restraining cell proliferation, particularly tumour-suppressor pathways associated with cell cycle inhibitors like TP53, RB, APC, p21, p27 and other cyclin-dependent kinase inhibitors [2]. Here, cannabinoids are relevant as several studies suggest that both CBD and THC can modulate these pathways and ultimately promote cell cycle arrest thus restoring growth-suppressive mechanisms.

In gastric cancer cells, CBD modulates the expression of p53 and upregulates p21 protein expression and the ataxia telangiectasia mutant (ATM) gene, inhibiting cyclin E and CDK2 expression, which results in cell cycle arrest in G0-1 phase, contributing to an antiproliferative effect [15]. Furthermore, in colorectal cancer cells that harbour wild-type p53, CBD triggers G0-1 cell cycle arrest, reduces CDK2 expression and engages in checkpoint signalling via p53 [67]. Therefore, CBD may reinforce tumour-suppressive cell cycle endpoints.

A recent study on prostate cancer using a synthetic cannabinoid, WIN-55, showed a dose-dependent antiproliferative effect on cell lines, with a proliferation reduction of up to 69% as well as a gain in cells in the G1 phase and a decrease in S phase, suggesting prostate cancer cell cycle arrest [68]. This supports the idea that cannabinoid-mediated antiproliferative effects may be partly explained by restoration of cell cycle control and inhibition of transition into DNA synthesis.

In oral cancer cells, modulation of the p53–p21 axis has been described following cannabinoid exposure, supporting a plausible function in cell-cycle restraint. Furthermore, THC on oral cancer cells inactivated the ERK1/2, Wnt, and NF-κB pathways, ultimately activating cyclin-dependent kinase inhibitory proteins such as p21 and p27, resulting in apoptosis [10].

The activation of the CB2R results in the expression of p27 and p21, regulating CDK and cyclin proteins and inhibiting the cell cycle, culminating in diminished cell proliferation [69]. All in all, this suggests cannabinoids may counteract growth suppressors inactivation while modulating p53-related checkpoint signalling, increasing the expression of p27 and p21 and inducing G0/1 cell cycle arrest across different tumour models.

## 11. Hallmark Enabling Characteristics

### 11.1. Loss of Genomic Integrity—Gene Mutations and CIN

The accumulation of genetic and chromosomal changes that drive cancer development is known as loss of genomic integrity, including point mutations, copy-number alterations, chromosomal instability and DNA repair defects, laying the foundation for cancer as a chronically expansive disease [2]. The relation between cannabis and genomic integrity is still poorly defined with some suggesting CBD may induce DNA damage and THC-related cell cycle block contributing to DNA damage accumulation and apoptosis.

To date, few studies are available regarding the potential for the use of cannabinoids to damage DNA. Nonetheless, CBD has been found to induce the formation of micronuclei in mouse bone marrow cells and could potentially result in chromosome aberrations. In addition, low doses of frequently used CBD could result in DNA chromosomal damage in human-derived cells, contrary to its antitumoural effects, contrasting with its described antitumoural effects in other models [70]. Following CBD exposure, nuclear hypodiploidy and DNA strand breaks have been observed [13]. As such, although CBD has been studied for its anticancer potential, the possible genotoxic effects should also be considered.

THC blocks oral cancer cells at the G2/M phase, through inhibition of Cdc2 dephosphorylation, which can lead to irreparable DNA damage, genomic instability and ultimately apoptosis [10]. This may represent a dual effect as accumulation of DNA damage and genomic instability can contribute to tumour cell death while also raising the need to better understand whether cannabis-induced genomic damage is restricted to cancer cells. Though evidence regarding cannabis and genomic integrity remains highly limited, it might influence DNA damage and chromosomal alterations, thus requiring further clarification.

### 11.2. Non-Mutational Epigenetic Reprogramming

Changes in gene expression can occur with no changes in DNA, through non-mutational epigenetic reprogramming namely DNA methylation and histone alteration or even transcriptional regulation, contributing to several cancer-related characteristics such as tumour heterogeneity, phenotypic plasticity, metastatic adaptation and therapy resistance, ultimately contributing to the viability of tumour cells [2]. In this context, cannabinoids may be relevant and have been shown to modulate pathways with epigenetic regulatory capacity, particularly through NF-κB signalling and DNA methylation-related mechanisms.

NF-κB signalling can produce epigenetic modifications after its translocation to the nucleus, modulating histone acetylation and methylation. Generally studies have found that CBD inhibits NF-κB’s nuclear translocation, in contrast with THC and WIN-55 which enhance this translocation, ultimately having the ability to produce epigenetic alterations [16]. This suggests different cannabinoids might have divergent effects on epigenetic regulation.

In GBM cancer cells, CBD was shown to induce DNA methylation on the CpG islands, thus potentially interfering with the expression of DNA repair enzymes like MGMT through promoter hypermethylation, showing a novel epigenetic mechanism underpinning CBD’s synergy with TMZ [63]. This is particularly relevant as MGMT promoter methylation closely associates with TMZ sensitivity in GBM, suggesting that CBD-induced epigenetic modulation can contribute to treatment response.

Overall, even though evidence remains limited cannabis may influence epigenetic reprogramming with implications for tumour progression and response to therapy.

### 11.3. Tumour-Promoting Inflammation

Chronic inflammatory responses support tumour initiation, progression, invasion, tumour evasion and ultimately resistance to cell death. Although inflammation can contribute to antitumour activity, persistent activation of immune and stromal cells in the TME can cause the opposite and promote cancer progression [2]. In this setting, CBD has shown immunosuppressive effects by regulating different signalling pathways and cytokine expression.

Inflammation is closely related to cancer development, with cells like tumour-associated mast cells, T and B lymphocytes, macrophages, dendritic cells (DCs), neutrophils, and MDSCs, associating with tumour-promoting inflammation [2]. These cells may contribute to tumour progression by producing cytokines, chemokines and growth factors, sustaining immune suppression and tissue remodelling.

CBD has been shown to inhibit the expression of COX-2 (cyclooxygenase type 2) in human neutrophils at therapeutically relevant doses [71]. Treatment with CBD also led to an important decrease of chemotactic agents such as IL-1β, CXCL9 and CXCL10, resulting in the suppression of inflammatory macrophage infiltration [72], suggesting that CBD may reduce tumour-promoting inflammation by limiting both inflammatory mediator production and recruitment of inflammatory immune cells into the TME.

On the other hand, greater NF-κB capacity in the TME associates with infiltration of several inflammatory cytokines, being essential for malignancy initiation, progression and inflammation-tumour transformation [73,74] CBD decreases the activated NF-κB p65 subunit while also downregulating the IL-1β and IL-6 transcription levels, inhibiting the stimulatory signals of NF-κB activation [75].

CBD also targets TLR3 and TLR4, therefore impeding the crosstalk with NF-κB and reducing IFN-β induction [76]. CBD suppresses the expression of the proinflammatory factor STAT1 and increases the anti-inflammatory factor STAT3, whose balance determines the outcome of the inflammation mediated by IFN-signalling [75,77]. CBD may as well boost the level of p53, implicitly affecting the transcriptional activity of NF-κB therefore regulating inflammatory cytokines and factors expression, as well as immune cells [48,78]. This suggests the regulation of tumour-promoting inflammation through several interconnected pathways.

In GBM, CBD was proven to have the capacity to interfere with the immunosuppressive TME by targeting P-selectin and IL-8 while hindering the immune checkpoint IDO and inhibiting cancer-associated fibroblasts activation and migration, therefore altering the TME’s protumourigenic balance [38,79].

On the whole, CBD may counteract tumour-promoting inflammation by reducing anti-inflammatory cytokines and chemokines, suppressing NF-κB’s activity and COX-2, modulating TLR signalling, as well as altering inflammatory and stromal components of the TME.

### 11.4. Innervation

The functional interaction between cancer cells and nerves includes peri- and intra-tumoural nerve fibres, neurotransmitter signalling, perineural invasion and, in some tumours, synaptic-like communication between neurons and cancer cells [2].

Cannabinoids’ role in cancer-related innervation is still in the early phase of understanding. Nonetheless, in other disease-related models involving neural dysfunction and synaptic signalling, cannabis has shown promising results. In fact, a study focused on the role of CBD following traumatic brain injury (TBI) showed that CBD regulates abnormal glutamate signal transmission in astrocytes. CBD effectively reduced astrocyte TNF-α secretion, decreasing neurotransmitter release and initiating synaptic remodelling, offering a potential therapeutic option for TBI-related synaptic dysfunction [80]. Although this evidence is not cancer-specific, it suggests that CBD may modulate astrocyte-mediated neurotransmission and synaptic remodelling, which are increasingly relevant in cancer neuroscience.

A study in retinoblastoma (Rb) cells showed that in etoposide-resistant cells there is a lower expression of transient receptor potential melastatin 8 (TRPM8) and cannabinoid receptor agonist 1 (CNR1) compared to responsive cells. The crosstalk between NGF, CB1/CNR1, and TRPM8 appears to modulate calcium signalling dynamics, contributing to the differences in etoposide sensitivity, based on this neurotropic signalling [81]. This suggests that cannabinoid-related receptors may interact with neurotrophic and calcium-dependent pathways, potentially influencing tumour cell behaviour and treatment response in neural-derived cancers.

Another study with human SHSY5Y neuroblastoma cells showed that these cells constitutively produce 2-AG, acting in an autocrine manner to promote neuritic outgrowth, highlighting a role for endocannabinoid signalling in neural process extension and tumour-associated innervation-related biology [82]. Overall, even though direct evidence connecting cannabinoids with cancer innervation is scarce, these findings suggest cannabinoid and endocannabinoid signalling might influence neural features important in cancer biology.

### 11.5. Polymorphic Microbiomes

Polymorphic microbiomes are highly variable communities of bacteria, fungi, viruses and even archaea and protozoa that colonise different tissues, influencing not only cancer development but also the response to different treatments. The gut microbiome is the best studied, but other microbiomes may also modulate different cancer hallmarks, particularly immune evasion and tumour-promoting inflammation and genomic instability [2]. Here, cannabis can be relevant as it interacts with the gut microbiome and the endocannabinoid system, while the composition of the microbiota can also influence cannabinoid metabolism and activity.

The gut microbiota is highly mixed and represents a dynamic population of microbes in the gastrointestinal tract, essential for several physiological processes and its role in cancer is now recognised. Dysbiosis may influence inflammation, immune regulation, epithelial barrier function and response to therapy, all of which may affect tumour behaviour [83].

The activation of CB1R can inhibit neurotransmitter release in the enteric system, resulting in reduced GI motility and secretion, as opposed to CB2R activation in the gut mucosa, which regulates the gut immune homeostasis [84].

Emerging evidence suggests that the anti-cancer effects of cannabis can be modulated by the gut microbiome, as the microbiota can metabolise cannabis and thus potentially influence its bioavailability and pharmacological activity. Furthermore, gut microbiome composition may influence the concentration and activity of cannabis in the GI tract and result in different outcomes [83]. The microbiota has β-glucuronidase which deconjugates THC’s metabolites, resulting in the release of its active form in the circulation [83]. Gut microbiota metabolites like short-chain fatty acids (SCFAs) interact with the endocannabinoid system and regulate the expression of cannabinoid receptors, affecting gut inflammation and motility [85]. These mechanisms suggest that microbiome composition may be an important determinant of cannabinoid exposure and downstream biological effects.

On the other hand, chronic exposure to THC can lead to gut microbiota changes and has been connected to an increase in *Akkermansia muciniphila* in mice, related to an improved gut barrier role and metabolic health. In contrast, in the human gut, cannabis use has been associated with an increase in *Bacteroides* species, related to inflammation and metabolic disorders [83]. Gut bacteria-derived metabolites such as secondary bile acids, can also activate cannabinoid receptors, suggesting that cannabis may reshape the gut microbiome in different ways depending on host, dose, exposure pattern and microbial baseline composition.

Gut microbiota-cannabis interaction therefore bidirectional, with each component dynamically influencing the other.

## 12. Discussion

**Figure 1:**
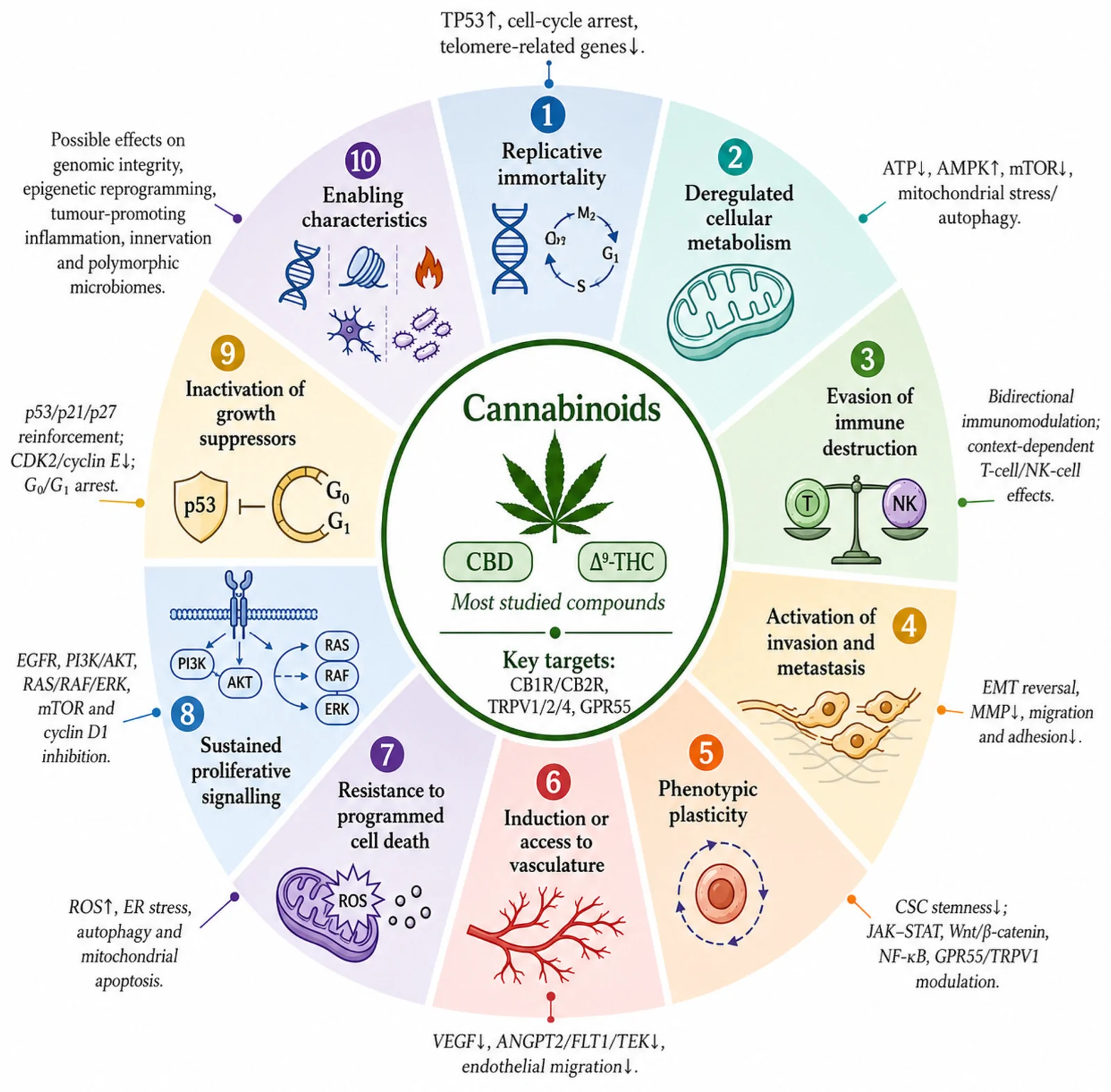
Graphical summary of the potential influence of cannabinoids across the hallmarks of cancer, summarizing the reported effects of cannabinoids across cancer hallmarks and enabling characteristics. The surrounding annotations indicate the principal molecular and cellular mechanisms described in the reviewed studies. Upward and downward arrows indicate reported increases and decreases, respectively. Figure created by the authors, based on the evidence reviewed in this manuscript.

Cannabinoids can interfere with several biological mechanisms involved in cancer development and progression and even though their role in oncology remains mostly supportive with predominance in symptom control, preclinical studies increasingly suggest that cannabis can also influence tumour biology. When looked upon through the several cancer characteristic hallmarks, cannabinoids appear to affect multiple and overlapping pathways from proliferation and metabolism to immune regulation and microenvironment modulation (Figure 1) [3,4,5].

One of the most consistent findings is CBD’s antiproliferative effect in several tumour models, shown to downregulate EGFR and inhibit signalling via PI3K/AKT, RAS/RAF/ERK and cyclin D1, which are central pathways with a prominent role in sustaining proliferative signalling [5,12,61]. At the same time, cannabis appears to also counteract the inactivation of growth suppressors via modulation of p53, p21 and p27-related pathways, while also diminishing CDK2 and cyclin E expression and inducing G0/1 cell cycle arrest [5,16,67,78]. As such, cannabinoids appear to affect tumour proliferation by simultaneously inhibiting positive growth signals and reinforcing mechanisms of cell-cycle restraint.

While affecting TP53, cell cycle arrest and telomere-related genes, CBD appears to interfere with cancer cells’ replicative immortality. In fact, in breast cancer cell models, CBD increased the levels of TP53 and induced G0/1 and S-phase arrest while decreasing the expression of telomere function and structure related genes, implying a limitation of replication capacity of tumour cells by cannabinoids [5,16,17].

Programmed cell death induction is yet another important mechanism described across several tumour types and also appears to be heavily influenced by cannabinoids. In fact, CBD increases ROS production and upregulates NADPH oxidases, contributing also to mitochondrial dysfunction and activation of apoptotic effectors [5]. Furthermore, in HCC, GBM and endometrial cancer models, cannabinoids induced mitochondrial-mediated apoptosis and ER stress, ultimately contributing to cell death. At the same time, these mechanisms are also closely linked to cancer metabolism given that cannabis can reduce ATP levels, inhibit mTOR and disrupt mitochondrial respiration, thus “forcing” cancer cells into energetic stress and promoting ROS-mediated apoptosis [7,9,18,19,55,62,63].

On the other hand, cannabinoids also influence invasion, metastasis and tumour vasculature through several pathways and mechanisms. CBD inhibits MMP-related extracellular matrix degradation while also reversing EMT markers and increasing E-cadherin expression, as well as blocking Wnt/β-catenin, IL-1β/IL-1R/β-catenin and GPR55-related pathways, thus contributing to a reduction in tumour cell migration, endothelial adhesion and ultimately diminishing metastasis [5,23,47,49,51]. At the same time, CBD has shown relevant antiangiogenic effects by suppressing HUVEC migration and reducing the expression of angiogenesis-related genes like *ANGPT2*, *FLT1* and *TEK*, while also reducing VEGF, MMP2, MMP9 and endothelin-1, fundamentally decreasing cancer cells survival by reducing their vascular supply [25,46,51,59].

Conversely, the interaction between the immune system and cannabinoids appears to be more complex. CB2R is wildly expressed in immune cells, making cannabinoids able to modulate T cells, dendritic cells, macrophages, MDSCs, NK cells and also the production of cytokines. While CBD and THC produce immunosuppressive effects, such as reducing T-cells’ activation while triggering MDSCs and suppression of inflammatory cytokines, in selected models immunostimulatory effects were also seen, namely increased NK-cells and improved T-cell proliferation and inhibition of IDO, suggesting not only a bidirectional but also highly context-dependent effect [31,38,72,73,74,75,79].

Furthermore, CBD also appears to affect cancer cells’ phenotypic plasticity through its effects on CSCs, as it’s been shown to regulate JAK-STAT, Wnt/β-catenin, NF-κB, CD44, GPR55 and TRPV1-related pathways and even to reduce the formation of neurospheres in GBM cells, thus affecting stemness-related mechanisms [7,50,52,53,55,56]. At the same time, given its association with micronuclei formation and chromosomal damage in some cell models, the role of CBD in genomic integrity has also been questioned, as it may influence epigenetic reprogramming through NF-κB modulation and DNA methylation, particularly MGMT promoter methylation in GBM [10,23,70,86]. This represents a context-dependent paradox, as cannabinoid-induced DNA damage may contribute to tumour-cell arrest or death, while its potential effects on non-malignant cells and the resulting therapeutic window remain insufficiently characterised [70]. Moreover, cannabis also has a bidirectional relationship with microbiomes, possibly modulating gut immune homeostasis and neurotransmission [83,84].

Current evidence supports preliminary and context-dependent pharmacodynamic interactions between CBD and THC in selected preclinical cancer models [64,87]. However, these findings have not been consistently reproduced in vivo [64] and do not establish a clinically validated entourage effect involving the broader phytochemical composition of *Cannabis sativa* [88]. Additionally, although direct tumour–normal comparisons remain limited, several studies suggest relative cannabinoid selectivity toward malignant cells in breast, glioblastoma and ovarian cancer models [16,21,65]. However, this evidence remains preclinical and insufficient to establish a consistent therapeutic window.

Despite these promising findings, current evidence is predominantly preclinical with most studies relying on established cell lines, murine/xenograft models and mainly focus on CBD and a restricted number of tumours. Additionally, there is still a lack of evidence regarding the correspondence between the cannabinoid in vitro dosage and the in vivo necessary dose to achieve its antitumoural effect. However, studies are ongoing evaluating the effects of cannabinoids as combination treatments with conventional chemotherapy in patients with specific cancer types [89]. In several cellular studies, the concentrations required to produce antitumour effects may not correspond to clinically achievable exposures, limiting the direct translation of these findings into human oncology. Furthermore, differences in cannabinoid receptor expression, tumour subtype, immune competence and characteristics of the experimental models may further explain some of the contradictory findings reported across studies. Given the heterogeneity of cannabinoid formulations, concentrations and administration routes, comparison between studies may not be clinically achievable. Important gaps remain regarding tumour selectivity, optimal dosing, pharmacokinetics, long-term safety and interactions with anticancer therapies, highlighting the need for standardised preclinical models and well-designed translational and clinical studies to determine whether the molecular effects described can result in clinically meaningful antitumour activity.

## 13. Conclusions

Cannabinoids have demonstrated biologically plausible promising antitumour effects by interacting with several cancer hallmarks, including tumour angiogenesis, resistance to programmed cell death, metastasis and invasion, as well as immune evasion and phenotypic plasticity. Among cannabinoid compounds, CBD has been the most studied and has consistently shown promising antiproliferative, pro-apoptotic and antiangiogenic properties in preclinical models, highlighting its potential as a therapeutic adjunct in oncology, contributing to better cancer-related outcomes. Nonetheless, the molecular mechanisms underlying the anticancer effects of cannabinoids remain incompletely understood. Their biological activity appears to depend on multiple factors, including the cannabinoid investigated, receptor expression, tumour type and molecular subtype, as well as interactions with the microbiome and TME. Moreover, even though several studies suggest potential and promising antitumour mechanisms of cannabis, the available evidence remains predominantly preclinical and heterogeneous, warranting cautious interpretation.

Future research should focus on elucidating the pharmacokinetic and pharmacodynamic properties of cannabinoids, optimising dosing strategies, and evaluating their potential role in combination with established anticancer therapies. Well-designed translational studies and adequately powered clinical trials are fundamental to understand if the encouraging findings noted in experimental models translate into meaningful clinical benefit. Addressing these challenges will be critical to defining the therapeutic role of cannabinoids within multimodal cancer treatment.

## Figures and Tables

**Table 1:** Summary of the potential influence of cannabinoids on the hallmarks of cancer.

Hallmark of Cancer	Potential Influence of Cannabinoids
Replicative Immortality	Potential increase in TP53 expression, inducing G0/G1 and S-phase cell-cycle arrest; Downregulation of genes involved in telomere structure and function, potentially limiting the replicative capacity of cancer cells; Modulation of FOXM1 and GDF15 may further restrict aberrant proliferation and promote differentiation.
Deregulation of Cellular Metabolism	Cannabinoids may reduce intracellular ATP levels, activate AMPK and inhibit mTOR, inducing energetic stress and autophagy; CBD and THC can disrupt mitochondrial respiration, calcium homeostasis and REDOX balance.
Evasion of Immune Destruction	CBD and THC may suppress T-cell activation, induce MDSCs and reduce inflammatory cytokine production; Selected models also show increased NK-cell activity, T-cell proliferation, antigen presentation and IDO inhibition; Cannabinoid immunomodulation appears bidirectional, dose-dependent and highly context-specific.
Activation of Invasion and Metastasis	CBD may reverse EMT, increase E-cadherin and inhibit Wnt/β-catenin, IL-1β/AKT and GPR55-related signalling; CBD may reduce MMP-mediated extracellular matrix degradation and tumour–endothelial adhesion; Modulation of ID-1, PAI-1 and other pro-metastatic mediators may further limit migration and metastatic dissemination.
Phenotypic Plasticity	CBD may target CSC survival and stemness through JAK–STAT, Wnt/β-catenin, NF-κB, CD44, GPR55 and TRPV1-related pathways; CBD may reduce neurosphere formation and promote CSC differentiation in GBM; Disruption of interactions between CSCs and the TME may affect tumour recurrence, adaptation and treatment resistance.
Induction or Access to Vasculature	CBD may reduce endothelial-cell migration and the expression of VEGF and angiogenesis-related genes, including *ANGPT2*, *FLT1* and *TEK*; Modulation of TIMP-1, MMPs and proangiogenic mediators may impair extracellular matrix remodelling; These mechanisms may interfere with the formation and maintenance of tumour vasculature.
Resistance to Programmed Cell Death	CBD may increase ROS production, disrupt mitochondrial function and activate BAX, caspase-3 and caspase-9 while reducing BCL-2; Cannabinoids may also promote ER stress and autophagy; These overlapping mechanisms may counteract tumour resistance to apoptosis and other forms of programmed cell death.
Sustained Proliferative Signalling	CBD may inhibit EGFR, PI3K/AKT, RAS/RAF/ERK, mTOR and cyclin D1 signalling; These effects may reduce tumour-cell proliferation and colony formation while inducing cell-cycle arrest; Cannabinoids may also increase sensitivity to conventional anticancer treatments.
Inactivation of Growth Suppressors	CBD and THC may modulate p53 and increase the expression of the cyclin-dependent kinase inhibitors p21 and p27; CBD may reduce CDK2 and cyclin E expression; These effects may promote G0/G1 arrest and partially restore growth-suppressive cell-cycle control.
Hallmark Enabling Characteristics	**Loss of genomic integrity—Gene mutations and CIN** CBD has been associated with micronucleus formation, chromosomal aberrations and DNA strand breaks, while THC-induced G2/M arrest may promote DNA-damage accumulation; The selectivity of these effects for cancer cells remains unclear.
	**Non-mutational epigenetic reprogramming** Cannabinoids may differentially regulate NF-κB nuclear translocation and DNA methylation, with CBD potentially promoting MGMT promoter methylation and increasing TMZ sensitivity in GBM.
	**Tumour-promoting inflammation:** CBD may reduce COX-2 and NF-κB activity, inflammatory cytokines and chemokines, TLR signalling and the activation of stromal components within the TME.
	**Innervation** Direct cancer-specific evidence remains limited; However cannabinoid signalling may influence astrocyte-mediated neurotransmission, neurotrophic and calcium-dependent pathways and neurite formation.
	**Polymorphic microbiomes:** Cannabinoids and the gut microbiome interact bidirectionally, as microbial composition may alter cannabinoid metabolism and bioavailability, while cannabinoid exposure may modify microbial communities, inflammation and endocannabinoid signalling.

AKT, protein kinase B; AMPK, AMP-activated protein kinase; ANGPT2, angiopoietin 2; ATP, adenosine triphosphate; BAX, BCL2-associated X protein; BCL-2, B-cell lymphoma 2 protein; CBD, cannabidiol; CDK2, cyclin-dependent kinase 2; CIN, chromosomal instability; COX-2, cyclooxygenase-2; CSC, cancer stem cell; EGFR, epidermal growth factor receptor; EMT, epithelial–mesenchymal transition; ER, endoplasmic reticulum; ERK, extracellular signal-regulated kinase; FLT1, Fms-related receptor tyrosine kinase 1; FOXM1, forkhead-box M1; GBM, glioblastoma; GDF15, growth differentiation factor-15; GPR55, G-protein-coupled receptor-55; ID-1, inhibitor of DNA binding 1; IDO, indoleamine 2,3-dioxygenase; IL-1β, interleukin-1 beta; JAK–STAT, Janus kinase–signal transducer and activator of transcription; MDSC, myeloid-derived suppressor cell; MGMT, O6-methylguanine-DNA methyltransferase; MMP, matrix metalloproteinase; mTOR, mechanistic target of rapamycin; NF-κB, nuclear factor kappa-light-chain-enhancer of activated B cells; NK, natural killer; PAI-1, plasminogen activator inhibitor 1; PI3K, phosphoinositide 3-kinase; RAF, rapidly accelerated fibrosarcoma; RAS, rat sarcoma; ROS, reactive oxygen species; TEK, TEK receptor tyrosine kinase; THC, tetrahydrocannabinol; TIMP-1, tissue inhibitor of metalloproteinases-1; TLR, Toll-like-receptor; TME, tumour microenvironment; TMZ, temozolomide; TP53, tumour protein p53 gene; TRPV1, transient receptor potential vanilloid 1; VEGF, vascular endothelial growth factor.

## Data Availability

No new data were created or analysed in this study. Data sharing is not applicable to this article.

## Abbreviations

The following abbreviations are used in this manuscript:

2-AG	2-Arachidonoylglycerol
AKT	Protein kinase B
AML1a	Acute myeloid leukaemia 1a
AMP	Adenosine monophosphate
AMPK	AMP-activated protein kinase
ANGPT2	Angiopoietin 2
AP-1	Activator protein 1
APAF1	Apoptotic peptidase activating factor 1
APC	APC regulator of WNT signalling
ASK1	Apoptosis signal-regulating kinase 1
ATF	Activating transcription factor
ATM	Ataxia-telangiectasia mutated
ATP	Adenosine triphosphate
BAD	BCL2-associated agonist of cell death
BAX	BCL2-associated X protein
BCL-2	B-cell lymphoma 2 protein
BCL2	BCL2 apoptosis regulator gene
BID	BH3-interacting domain death agonist
BiP	Binding immunoglobulin protein
BIRC3	Baculoviral IAP repeat containing 3
BRAF	B-Raf proto-oncogene
CASP3	Caspase 3
CASP8	Caspase 8
CASP9	Caspase 9
CB	Cannabinoid receptor
CB1R	Cannabinoid receptor type 1
CB2R	Cannabinoid receptor type 2
CBD	Cannabidiol
CCND1	Cyclin D1
CD103	Cluster of differentiation 103
CD44	Cluster of differentiation 44
CD8+	Cluster of differentiation 8-positive
Cdc2	Cell division cycle 2
CDK	Cyclin-dependent kinase
CDK1	Cyclin-dependent kinase 1
CDK2	Cyclin-dependent kinase 2
CHOP	CCAAT/enhancer-binding protein homologous protein
CIK	Cytokine-induced killer
CIN	Chromosomal instability
CNR1	Cannabinoid receptor 1 gene
COX-2	Cyclooxygenase-2
CpG	Cytosine-phosphate-guanine dinucleotide
CSC	Cancer stem cell
CXCL	C-X-C motif chemokine ligand
CXCL10	C-X-C motif chemokine ligand 10
CXCL16	C-X-C motif chemokine ligand 16
CXCL8	C-X-C motif chemokine ligand 8; interleukin-8
CXCL9	C-X-C motif chemokine ligand 9
DCs	Dendritic cells
Δ9-THC	Delta-9-tetrahydrocannabinol
DNA	Deoxyribonucleic acid
EGF	Epidermal growth factor
EGFR	Epidermal growth factor receptor
EMT	Epithelial-mesenchymal transition
ER	Endoplasmic reticulum
ERK	Extracellular signal-regulated kinase
ERK1/2	Extracellular signal-regulated kinases 1 and 2
ET-1	Endothelin-1
FLT1	Fms-related receptor tyrosine kinase 1
FOXM1	Forkhead box M1
GBM	Glioblastoma
GDF15	Growth differentiation factor 15
GPCR	G protein-coupled receptor
GPR55	G protein-coupled receptor 55
HCC	Hepatocellular carcinoma
HER2	Human epidermal growth factor receptor 2
HIF-1α	Hypoxia-inducible factor 1 alpha
HUVEC	Human umbilical vein endothelial cell
ICAM-1	Intercellular adhesion molecule 1
ID-1	Inhibitor of DNA binding 1
IDO1	Indoleamine 2,3-dioxygenase 1
IFN-β	Interferon beta
IFN-γ	Interferon gamma
IL-10	Interleukin-10
IL-17	Interleukin-17
IL-1β	Interleukin-1 beta
IL-1R	Interleukin-1 receptor
IL-6	Interleukin-6
IL-8	Interleukin-8
IRE1	Inositol-requiring enzyme 1
IRE1α	Inositol-requiring enzyme 1 alpha
JAK	Janus kinase
JAK-STAT	Janus kinase-signal transducer and activator of transcription
JNK	c-Jun N-terminal kinase
JNK1/2	c-Jun N-terminal kinases 1 and 2
KDR	Kinase insert domain receptor
KRAS	KRAS proto-oncogene, GTPase
LC3-II	Lipidated form II of microtubule-associated protein 1 light chain 3
LPI	Lysophosphatidylinositol
MAPK	Mitogen-activated protein kinase
MDSC	Myeloid-derived suppressor cell
MEK	Mitogen-activated protein kinase/extracellular signal-regulated kinase
MGMT	O6-methylguanine-DNA methyltransferase
MMP	Matrix metalloproteinase
MMP-2	Matrix metalloproteinase 2 protein
MMP-9	Matrix metalloproteinase 9 protein
MMP2	Matrix metalloproteinase 2 gene
MMP9	Matrix metalloproteinase 9 gene
MMPs	Matrix metalloproteinases
mTOR	Mechanistic target of rapamycin
MYC	MYC proto-oncogene
NADPH	Nicotinamide adenine dinucleotide phosphate
NF-κB	Nuclear factor kappa-light-chain-enhancer of activated B cells
NGF	Nerve growth factor
NK	Natural killer
NKT	Natural killer T
NOX4	NADPH oxidase 4
NOXA	Pro-apoptotic NOXA protein; encoded by PMAIP1
NRF2	Nuclear factor erythroid 2-related factor 2
p21	Cyclin-dependent kinase inhibitor 1A
p22phox	Phagocyte oxidase p22 subunit
p27	Cyclin-dependent kinase inhibitor 1B
p38 MAPK	p38 mitogen-activated protein kinase
p53	Tumour protein p53
p65	Nuclear factor κB p65 subunit
PAI-1	Plasminogen activator inhibitor 1
PANC-1	Human pancreatic carcinoma cell line PANC-1
PDGF-AA	Platelet-derived growth factor AA
PERK	Protein kinase R-like endoplasmic reticulum kinase
PHB	Prohibitin
PI3K	Phosphoinositide 3-kinase
PIK3CA	Phosphatidylinositol-4,5-bisphosphate 3-kinase catalytic subunit alpha
PPARγ	Peroxisome proliferator-activated receptor gamma
RAF	Rapidly accelerated fibrosarcoma
RAS	Rat sarcoma
RB	Retinoblastoma tumour suppressor protein
RB1	Retinoblastoma 1 tumour suppressor gene
ROS	Reactive oxygen species
SCFA	Short-chain fatty acid
SRC	SRC proto-oncogene
STAT	Signal transducer and activator of transcription
STAT1	Signal transducer and activator of transcription 1
STAT3	Signal transducer and activator of transcription 3
TBI	Traumatic brain injury
TEK	TEK receptor tyrosine kinase
THC	Tetrahydrocannabinol
TIMP-1	Tissue inhibitor of metalloproteinases 1
TLR	Toll-like receptor
TLR3	Toll-like receptor 3
TLR4	Toll-like receptor 4
TME	Tumour microenvironment
TMZ	Temozolomide
TNBC	Triple-negative breast cancer
TNF-α	Tumour necrosis factor alpha
TP53	Tumour protein p53 gene
TRP	Transient receptor potential
TRPM8	Transient receptor potential melastatin 8
TRPV	Transient receptor potential vanilloid
TRPV1	Transient receptor potential vanilloid 1
TRPV2	Transient receptor potential vanilloid 2
TRPV4	Transient receptor potential vanilloid 4
VEGF	Vascular endothelial growth factor
VEGFA	Vascular endothelial growth factor A
VHL	Von Hippel-Lindau tumour suppressor
WIN 55,212-2	Synthetic cannabinoid receptor agonist WIN 55,212-2
